# (*E*)-3-(3,4-Dimeth­oxy­phen­yl)-1-(2-hy­droxy­phen­yl)prop-2-en-1-one

**DOI:** 10.1107/S1600536811008361

**Published:** 2011-03-12

**Authors:** Jerry P. Jasinski, Ray J. Butcher, V. Musthafa Khaleel, B. K. Sarojini, H. S. Yathirajan

**Affiliations:** a Department of Chemistry, Keene State College, 229 Main Street, Keene, NH 03435-2001, USA; b Department of Chemistry, Howard University, 525 College Street NW, Washington, DC 20059, USA; c Department of Chemistry, P.A. College of Engineering, Mangalore 574 153, India; d Department of Studies in Chemistry, University of Mysore, Manasagangotri, Mysore 570 006, India

## Abstract

In the title compound, C_17_H_16_O_4_, the dihedral angle between the mean planes of the hy­droxy­phenyl and dimeth­oxy­phenyl rings is 5.9 (6)°. The mean plane of the prop-2-en-1-one group makes dihedral angles of 3.6 (0) and 2.6 (7)° with the hy­droxy­phenyl and dimeth­oxy­phenyl rings, respectively. An intra­molecular O—H⋯O hydrogen bond occurs. The crystal packing is stabilized by weak inter­molecular C—H⋯O contacts and π–π stacking inter­actions [centroid–centroid distance = 3.6571 (8) Å].

## Related literature

For related structures, see: Butcher *et al.* (2006 ▶); Cao *et al.* (2005 ▶); Harrison *et al.* (2007 ▶); Jasinski *et al.* (2010 ▶, 2011*a* ▶,*b* ▶); Ngaini *et al.* (2009 ▶); Radha Krishna *et al.* (2005 ▶); Sharma *et al.* (1997 ▶); Wu *et al.* (2005 ▶). For standard bond lengths, see: Allen *et al.* (1987 ▶).
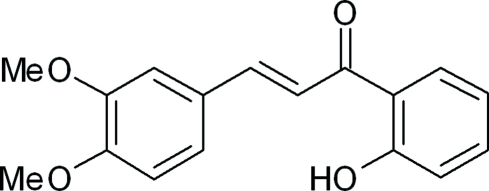


## Experimental

### 

#### Crystal data

C_17_H_16_O_4_
*M* *_r_* = 284.30
Monoclinic, 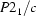

*a* = 14.2315 (2) Å
*b* = 8.0292 (1) Å
*c* = 13.6027 (2) Å
β = 110.0531 (14)°
*V* = 1460.11 (3) Å^3^
*Z* = 4
Cu *K*α radiation
μ = 0.76 mm^−1^
*T* = 295 K
0.51 × 0.47 × 0.35 mm

#### Data collection

Oxford Diffraction Gemini R diffractometer
Absorption correction: multi-scan (*CrysAlis RED*; Oxford Diffraction, 2007 ▶) *T*_min_ = 0.065, *T*_max_ = 1.000
6676 measured reflections
3014 independent reflections
2336 reflections with *I* > 2σ(*I*)
*R*_int_ = 0.021

#### Refinement

*R*[*F* ^2^ > 2σ(*F* ^2^)] = 0.048
*wR*(*F* ^2^) = 0.152
*S* = 1.12
3014 reflections
193 parameters
H-atom parameters constrained
Δρ_max_ = 0.18 e Å^−3^
Δρ_min_ = −0.18 e Å^−3^

### 

Data collection: *CrysAlis PRO* (Oxford Diffraction, 2007 ▶); cell refinement: *CrysAlis PRO*; data reduction: *CrysAlis RED* (Oxford Diffraction, 2007 ▶); program(s) used to solve structure: *SHELXS97* (Sheldrick, 2008 ▶); program(s) used to refine structure: *SHELXL97* (Sheldrick, 2008 ▶); molecular graphics: *SHELXTL* (Sheldrick, 2008 ▶); software used to prepare material for publication: *SHELXTL*.

## Supplementary Material

Crystal structure: contains datablocks global, I. DOI: 10.1107/S1600536811008361/kp2312sup1.cif

Structure factors: contains datablocks I. DOI: 10.1107/S1600536811008361/kp2312Isup2.hkl

Additional supplementary materials:  crystallographic information; 3D view; checkCIF report

## Figures and Tables

**Table 1:** Hydrogen-bond geometry (Å, °)

*D*—H⋯*A*	*D*—H	H⋯*A*	*D*⋯*A*	*D*—H⋯*A*
O1—H1*A*⋯O2	0.82	1.77	2.5021 (18)	147
C14—H14*A*⋯O2^i^	0.93	2.51	3.4250 (19)	170

Symmetry code: (i) 
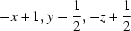
.

**Fig. 1.**
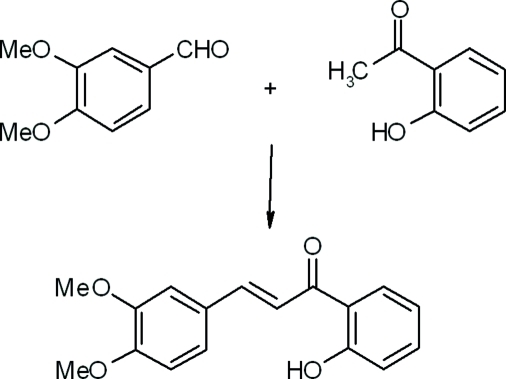
Reaction scheme for (I).

**Fig. 2.**
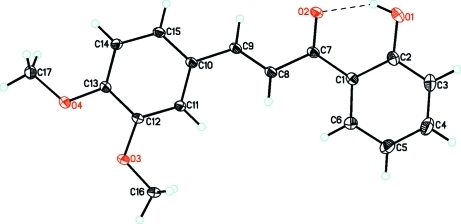
Molecular structure of the title compound showing the atom labeling scheme and 50% probability displacement ellipsoids. Dashed line indicates an O—H···O intramolecular bond.

**Fig. 3.**
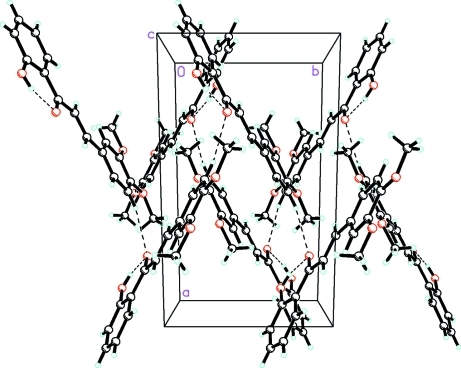
Packing diagram of the title compound viewed down the c axis. Dashed lines indicate O—H···O intramolecular hydrogen bonds and weak C—H···O intermolecular interactions.

## supplementary crystallographic information

### Comment

In continuation to our studies on crystal structures of chalcones (Jasinski *et al.*, 2010, 2011*a*, 2011*b*), we report here the synthesis (Fig. 1) and crystal structure of a new chalcone, C_17_H_16_O_4_, (I). The dihedral angle between the mean planes of the hydroxyphenyl and dimethoxyphenyl rings is 5.9 (6)° (Fig. 2). The mean plane of the prop-2-en-1-one group, the active site in this molecule, makes angles of 3.6 (0)° and 2.6 (7)°, respectively, with the hydroxyphenyl and dimethoxyphenyl rings.  Bond lengths and angles are normal (Allen *et al.*, 1987) and corespond to those observed in related compounds (Butcher *et al.*, 2006; Cao *et al.*, 2005; Harrison *et al.*, 2007; Jasinski *et al.*, 2010, 2011*a*, 2011*b*; Ngaini *et al.*, 2009; Radha Krishna *et al.*, 2005; Sharma *et al.*, 1997;  Wu *et al.*, 2005). Crystal packing is stabilized by O—H···O intramolecular hydrogen bonds, weak C—H···O intermolecular (Table 1) and π—π  stacking interactions (Table 2, Fig. 3).

### Experimental

2-Hydroxyacetophenone (1.36 g, 0.01 mol) was mixed with 3,4-dimethoxybenzaldehyde (1.66 g, 0.01 mol) and dissolved in ethanol (40 ml) (Fig. 1). To this solution, 5 mL of KOH (50%) was added at 278 K. The reaction mixture stirred overnight at room temperature and poured on to crushed ice. The pH of this mixture was adjusted to 3–4 with 2 *M* HCl aqueous solution. The resulting crude solid was filtered, washed successively with dilute HCl solution and distilled water and finally recrystallized from ethanol (95%) to give the pure chalcone. Crystals suitable for *X*-ray diffraction studies were grown by the slow evaporation of the solution of the compound in ethyl alcohol (m.p.: 378–381 K). Composition: Found (Calculated) for C_17_H_16_O_4_, C 75.25 (75.28); H: 5.98 (5.92).

### Refinement

The hydroxyl hydrogem (H1A) was located by a Fourier map, fixed at 0.82 Å and refined using the riding model. All of the remaining H atoms were placed in their calculated positions and then refined using the riding model with Atom—H lengths of 0.93Å (CH), 0.96Å (CH_3_). Isotropic displacement parameters for these atoms were set to 1.19–1.20 (CH), 1.49 (CH_3_) or 1.49 (OH) times *U*_eq_ of the parent atom.

### Crystal data

**Table:**

C_17_H_16_O_4_	*F*(000) = 600
*M**_r_* = 284.30	*D*_x_ = 1.293 Mg m^−^^3^
Monoclinic, *P*2_1_/*c*	Cu *K*α radiation, λ = 1.54184 Å
Hall symbol: -P 2ybc	Cell parameters from 4075 reflections
*a* = 14.2315 (2) Å	θ = 5.5–77.1°
*b* = 8.0292 (1) Å	µ = 0.76 mm^−^^1^
*c* = 13.6027 (2) Å	*T* = 295 K
β = 110.0531 (14)°	Prism, pale yellow
*V* = 1460.11 (3) Å^3^	0.51 × 0.47 × 0.35 mm
*Z* = 4	

### Data collection

**Table:**

Oxford Diffraction Gemini R diffractometer	3014 independent reflections
Radiation source: fine-focus sealed tube	2336 reflections with *I* > 2σ(*I*)
graphite	*R*_int_ = 0.021
Detector resolution: 10.5081 pixels mm^-1^	θ_max_ = 77.4°, θ_min_ = 6.4°
φ and ω scans	*h* = −17→16
Absorption correction: multi-scan (*CrysAlis RED*; Oxford Diffraction, 2007)	*k* = −9→9
*T*_min_ = 0.065, *T*_max_ = 1.000	*l* = −15→17
6676 measured reflections	

### Refinement

**Table:**

Refinement on *F*^2^	Primary atom site location: structure-invariant direct methods
Least-squares matrix: full	Secondary atom site location: difference Fourier map
*R*[*F*^2^ > 2σ(*F*^2^)] = 0.048	Hydrogen site location: inferred from neighbouring sites
*wR*(*F*^2^) = 0.152	H-atom parameters constrained
*S* = 1.12	*w* = 1/[σ^2^(*F*_o_^2^) + (0.0968*P*)^2^ + 0.0366*P*] where *P* = (*F*_o_^2^ + 2*F*_c_^2^)/3
3014 reflections	(Δ/σ)_max_ < 0.001
193 parameters	Δρ_max_ = 0.18 e Å^−^^3^
0 restraints	Δρ_min_ = −0.18 e Å^−^^3^

### Special details

**Table:**

Geometry. All e.s.d.'s (except the e.s.d. in the dihedral angle between two l.s. planes) are estimated using the full covariance matrix. The cell e.s.d.'s are taken into account individually in the estimation of e.s.d.'s in distances, angles and torsion angles; correlations between e.s.d.'s in cell parameters are only used when they are defined by crystal symmetry. An approximate (isotropic) treatment of cell e.s.d.'s is used for estimating e.s.d.'s involving l.s. planes.
Refinement. Refinement of *F*^2^ against ALL reflections. The weighted *R*-factor *wR* and goodness of fit *S* are based on *F*^2^, conventional *R*-factors *R* are based on *F*, with *F* set to zero for negative *F*^2^. The threshold expression of *F*^2^ > σ(*F*^2^) is used only for calculating *R*-factors(gt) *etc*. and is not relevant to the choice of reflections for refinement. *R*-factors based on *F*^2^ are statistically about twice as large as those based on *F*, and *R*- factors based on ALL data will be even larger.

### Fractional atomic coordinates and isotropic or equivalent isotropic displacement parameters (Å^2^)

**Table:**

	*x*	*y*	*z*	*U*_iso_*/*U*_eq_	
O1	0.88704 (12)	0.7981 (2)	0.17236 (10)	0.0876 (4)	
H1A	0.8351	0.7455	0.1617	0.131*	
O2	0.76002 (8)	0.62603 (18)	0.21587 (8)	0.0737 (4)	
O3	0.63965 (8)	0.32097 (14)	0.68696 (7)	0.0648 (3)	
O4	0.47076 (8)	0.18452 (14)	0.59076 (8)	0.0638 (3)	
C1	0.90537 (11)	0.71605 (18)	0.34900 (11)	0.0558 (3)	
C2	0.94152 (13)	0.7937 (2)	0.27531 (14)	0.0670 (4)	
C3	1.03495 (17)	0.8674 (3)	0.3086 (2)	0.0921 (6)	
H3A	1.0588	0.9173	0.2601	0.110*	
C4	1.09263 (17)	0.8676 (3)	0.4123 (2)	0.1082 (8)	
H4A	1.1553	0.9177	0.4338	0.130*	
C5	1.05822 (17)	0.7935 (3)	0.4855 (2)	0.1030 (8)	
H5A	1.0979	0.7934	0.5559	0.124*	
C6	0.96559 (14)	0.7203 (2)	0.45407 (14)	0.0744 (5)	
H6A	0.9425	0.6726	0.5038	0.089*	
C7	0.80752 (10)	0.63257 (19)	0.31157 (10)	0.0536 (3)	
C8	0.76620 (10)	0.55612 (18)	0.38547 (10)	0.0545 (3)	
H8A	0.8025	0.5598	0.4568	0.065*	
C9	0.67732 (10)	0.48135 (18)	0.35199 (10)	0.0531 (3)	
H9A	0.6448	0.4794	0.2799	0.064*	
C10	0.62489 (10)	0.40250 (17)	0.41423 (10)	0.0502 (3)	
C11	0.66334 (10)	0.40028 (17)	0.52433 (10)	0.0505 (3)	
H11A	0.7254	0.4481	0.5593	0.061*	
C12	0.61015 (10)	0.32804 (17)	0.58086 (10)	0.0497 (3)	
C13	0.51638 (10)	0.25396 (17)	0.52792 (10)	0.0501 (3)	
C14	0.47888 (10)	0.25475 (19)	0.41980 (11)	0.0553 (3)	
H14A	0.4173	0.2058	0.3845	0.066*	
C15	0.53281 (10)	0.3283 (2)	0.36387 (10)	0.0567 (4)	
H15A	0.5068	0.3279	0.2911	0.068*	
C16	0.73098 (14)	0.3979 (3)	0.74455 (12)	0.0755 (5)	
H16A	0.7843	0.3472	0.7271	0.113*	
H16B	0.7432	0.3845	0.8180	0.113*	
H16C	0.7276	0.5144	0.7278	0.113*	
C17	0.37373 (12)	0.1167 (2)	0.54210 (14)	0.0681 (4)	
H17A	0.3287	0.2033	0.5056	0.102*	
H17B	0.3505	0.0692	0.5944	0.102*	
H17C	0.3763	0.0318	0.4934	0.102*	

### Atomic displacement parameters (Å^2^)

**Table:**

	*U*^11^	*U*^22^	*U*^33^	*U*^12^	*U*^13^	*U*^23^
O1	0.1035 (10)	0.1039 (11)	0.0682 (8)	−0.0071 (8)	0.0458 (7)	0.0160 (7)
O2	0.0640 (6)	0.1134 (10)	0.0441 (5)	−0.0038 (6)	0.0193 (5)	0.0099 (5)
O3	0.0718 (6)	0.0810 (7)	0.0380 (5)	−0.0125 (5)	0.0142 (4)	0.0055 (4)
O4	0.0672 (6)	0.0759 (7)	0.0503 (5)	−0.0126 (5)	0.0226 (5)	0.0033 (5)
C1	0.0575 (7)	0.0584 (8)	0.0558 (8)	0.0092 (6)	0.0248 (6)	0.0038 (6)
C2	0.0745 (10)	0.0630 (9)	0.0739 (10)	0.0041 (7)	0.0389 (8)	0.0062 (7)
C3	0.0863 (13)	0.0863 (13)	0.1165 (18)	−0.0127 (10)	0.0514 (13)	0.0123 (12)
C4	0.0760 (13)	0.1022 (16)	0.141 (2)	−0.0257 (11)	0.0305 (14)	0.0055 (15)
C5	0.0775 (12)	0.1164 (18)	0.0948 (15)	−0.0212 (12)	0.0034 (11)	0.0067 (13)
C6	0.0695 (9)	0.0837 (11)	0.0647 (10)	−0.0050 (8)	0.0159 (8)	0.0055 (8)
C7	0.0538 (7)	0.0663 (8)	0.0440 (6)	0.0109 (6)	0.0210 (5)	0.0039 (6)
C8	0.0574 (7)	0.0671 (8)	0.0416 (6)	0.0069 (6)	0.0205 (5)	0.0025 (6)
C9	0.0590 (7)	0.0632 (8)	0.0407 (6)	0.0082 (6)	0.0218 (5)	0.0010 (6)
C10	0.0552 (7)	0.0572 (7)	0.0408 (6)	0.0063 (5)	0.0197 (5)	−0.0008 (5)
C11	0.0523 (7)	0.0570 (7)	0.0417 (6)	−0.0003 (5)	0.0154 (5)	−0.0014 (5)
C12	0.0572 (7)	0.0530 (7)	0.0377 (6)	0.0026 (5)	0.0145 (5)	0.0009 (5)
C13	0.0547 (7)	0.0526 (7)	0.0452 (7)	0.0027 (5)	0.0201 (5)	0.0008 (6)
C14	0.0495 (6)	0.0680 (8)	0.0471 (7)	−0.0012 (6)	0.0147 (5)	−0.0056 (6)
C15	0.0568 (7)	0.0755 (9)	0.0364 (6)	0.0049 (6)	0.0143 (5)	−0.0027 (6)
C16	0.0726 (10)	0.1061 (14)	0.0400 (7)	−0.0133 (9)	0.0094 (6)	−0.0028 (8)
C17	0.0642 (9)	0.0744 (10)	0.0710 (10)	−0.0079 (7)	0.0300 (7)	−0.0029 (8)

### Geometric parameters (Å, °)

**Table:**

O1—C2	1.349 (2)	C8—C9	1.331 (2)
O1—H1A	0.8200	C8—H8A	0.9300
O2—C7	1.2454 (16)	C9—C10	1.4517 (19)
O3—C12	1.3591 (15)	C9—H9A	0.9300
O3—C16	1.4094 (19)	C10—C15	1.388 (2)
O4—C13	1.3578 (16)	C10—C11	1.4073 (17)
O4—C17	1.4193 (19)	C11—C12	1.3784 (19)
C1—C6	1.392 (2)	C11—H11A	0.9300
C1—C2	1.418 (2)	C12—C13	1.4121 (19)
C1—C7	1.470 (2)	C13—C14	1.3820 (18)
C2—C3	1.382 (3)	C14—C15	1.384 (2)
C3—C4	1.367 (3)	C14—H14A	0.9300
C3—H3A	0.9300	C15—H15A	0.9300
C4—C5	1.386 (4)	C16—H16A	0.9600
C4—H4A	0.9300	C16—H16B	0.9600
C5—C6	1.371 (3)	C16—H16C	0.9600
C5—H5A	0.9300	C17—H17A	0.9600
C6—H6A	0.9300	C17—H17B	0.9600
C7—C8	1.4623 (19)	C17—H17C	0.9600
			
C2—O1—H1A	109.5	C15—C10—C11	118.47 (12)
C12—O3—C16	117.53 (12)	C15—C10—C9	119.11 (12)
C13—O4—C17	117.57 (11)	C11—C10—C9	122.41 (12)
C6—C1—C2	118.03 (15)	C12—C11—C10	120.76 (12)
C6—C1—C7	122.90 (14)	C12—C11—H11A	119.6
C2—C1—C7	119.06 (13)	C10—C11—H11A	119.6
O1—C2—C3	118.35 (17)	O3—C12—C11	125.54 (12)
O1—C2—C1	121.78 (15)	O3—C12—C13	114.69 (12)
C3—C2—C1	119.88 (18)	C11—C12—C13	119.77 (12)
C4—C3—C2	120.5 (2)	O4—C13—C14	125.33 (13)
C4—C3—H3A	119.7	O4—C13—C12	115.10 (11)
C2—C3—H3A	119.7	C14—C13—C12	119.56 (12)
C3—C4—C5	120.4 (2)	C13—C14—C15	120.15 (13)
C3—C4—H4A	119.8	C13—C14—H14A	119.9
C5—C4—H4A	119.8	C15—C14—H14A	119.9
C6—C5—C4	119.9 (2)	C14—C15—C10	121.29 (12)
C6—C5—H5A	120.1	C14—C15—H15A	119.4
C4—C5—H5A	120.1	C10—C15—H15A	119.4
C5—C6—C1	121.2 (2)	O3—C16—H16A	109.5
C5—C6—H6A	119.4	O3—C16—H16B	109.5
C1—C6—H6A	119.4	H16A—C16—H16B	109.5
O2—C7—C8	119.91 (13)	O3—C16—H16C	109.5
O2—C7—C1	119.43 (13)	H16A—C16—H16C	109.5
C8—C7—C1	120.65 (12)	H16B—C16—H16C	109.5
C9—C8—C7	120.86 (12)	O4—C17—H17A	109.5
C9—C8—H8A	119.6	O4—C17—H17B	109.5
C7—C8—H8A	119.6	H17A—C17—H17B	109.5
C8—C9—C10	127.97 (12)	O4—C17—H17C	109.5
C8—C9—H9A	116.0	H17A—C17—H17C	109.5
C10—C9—H9A	116.0	H17B—C17—H17C	109.5
			
C6—C1—C2—O1	−178.66 (16)	C8—C9—C10—C11	2.0 (2)
C7—C1—C2—O1	2.4 (2)	C15—C10—C11—C12	−0.9 (2)
C6—C1—C2—C3	1.4 (2)	C9—C10—C11—C12	178.26 (12)
C7—C1—C2—C3	−177.60 (16)	C16—O3—C12—C11	1.9 (2)
O1—C2—C3—C4	179.4 (2)	C16—O3—C12—C13	−177.80 (14)
C1—C2—C3—C4	−0.6 (3)	C10—C11—C12—O3	−179.11 (12)
C2—C3—C4—C5	0.1 (4)	C10—C11—C12—C13	0.6 (2)
C3—C4—C5—C6	−0.4 (4)	C17—O4—C13—C14	−4.4 (2)
C4—C5—C6—C1	1.1 (4)	C17—O4—C13—C12	176.45 (13)
C2—C1—C6—C5	−1.6 (3)	O3—C12—C13—O4	−1.09 (18)
C7—C1—C6—C5	177.29 (19)	C11—C12—C13—O4	179.19 (12)
C6—C1—C7—O2	−176.19 (15)	O3—C12—C13—C14	179.70 (13)
C2—C1—C7—O2	2.7 (2)	C11—C12—C13—C14	0.0 (2)
C6—C1—C7—C8	3.2 (2)	O4—C13—C14—C15	−179.36 (13)
C2—C1—C7—C8	−177.88 (13)	C12—C13—C14—C15	−0.2 (2)
O2—C7—C8—C9	−1.0 (2)	C13—C14—C15—C10	−0.1 (2)
C1—C7—C8—C9	179.55 (13)	C11—C10—C15—C14	0.6 (2)
C7—C8—C9—C10	−178.80 (13)	C9—C10—C15—C14	−178.54 (13)
C8—C9—C10—C15	−178.87 (14)		

### Hydrogen-bond geometry (Å, °)

**Table:**

*D*—H···*A*	*D*—H	H···*A*	*D*···*A*	*D*—H···*A*
O1—H1A···O2	0.82	1.77	2.5021 (18)	147
C14—H14A···O2^i^	0.93	2.51	3.4250 (19)	170

Symmetry codes: (i) −*x*+1, *y*−1/2, −*z*+1/2.

### Table 2 Selected geometric parmeters (Å): Cg···Cg π stacking interactions, Cg2 is the centroid of ring C10—C15 [Symmetry code: (i) 1-x, 1-y, 1-z]

**Table:**

*Cg*I···*Cg*J	Cg···Cg (Å)	CgI Perp (Å)	Cgj Perp (Å)	Slippage (Å)
Cg2···Cg2^i^	3.6571 (8)	3.5389 (6)	3.5389 (6)	0.92 (2)

## References

[bb1] Allen, F. H., Kennard, O., Watson, D. G., Brammer, L., Orpen, A. G. & Taylor, R. (1987). *J. Chem. Soc. Perkin Trans. 2*, pp. S1–19.

[bb2] Butcher, R. J., Yathirajan, H. S., Anilkumar, H. G., Sarojini, B. K. & Narayana, B. (2006). *Acta Cryst.* E**62**, o1633–o1635.

[bb3] Cao, D.-X., Li, G.-Z., Xue, G., Yu, W.-T. & Liu, Z.-Q. (2005). *Acta Cryst.* E**61**, o977–o979.

[bb4] Harrison, W. T. A., Kumari, V., Ravindra, H. J. & Dharmaprakash, S. M. (2007). *Acta Cryst.* E**63**, o2928. 10.1107/S0108270107021774 17609553

[bb5] Jasinski, J. P., Butcher, R. J., Chidan Kumar, C. S., Yathirajan, H. S. & Mayekar, A. N. (2010). *Acta Cryst.* E**66**, o2936–o2937. 10.1107/S1600536810041292 PMC3009291 21589107

[bb6] Jasinski, J. P., Butcher, R. J., Samshuddin, S., Narayana, B. & Yathirajan, H. S. (2011*b*). *Acta Cryst.* E**67**, o352–o353. 10.1107/S1600536811000353 PMC3051466 21523033

[bb7] Jasinski, J. P., Butcher, R. J., Siddaraju, B. P., Narayana, B. & Yathirajan, H. S. (2011*a*). *Acta Cryst.* E**67**, o313–o314. 10.1107/S1600536811000377 PMC3051752 21523001

[bb8] Ngaini, Z., Fadzillah, S. M. H., Hussain, H., Razak, I. A. & Fun, H.-K. (2009). *Acta Cryst.* E**65**, o1301–o1302. 10.1107/S1600536809017577 PMC2969631 21583159

[bb9] Oxford Diffraction (2007). *CrysAlis PRO* and *CrysAlis RED* Oxford Diffraction Ltd, Oxfordshire, England.

[bb10] Radha Krishna, J., Jagadeesh Kumar, N., Krishnaiah, M., Venkata Rao, C., Koteswara Rao, Y. & Puranik, V. G. (2005). *Acta Cryst.* E**61**, o1323–o1325.

[bb11] Sharma, N. K., Kumar, R., Parmar, V. S. & Errington, W. (1997). *Acta Cryst.* C**53**, 1438–1440.

[bb12] Sheldrick, G. M. (2008). *Acta Cryst.* A**64**, 112–122. 10.1107/S0108767307043930 18156677

[bb13] Wu, H., Xu, Z. & Liang, Y.-M. (2005). *Acta Cryst.* E**61**, o1434–o1435.

